# Detection of duo-schistosome infection from filtered urine samples from school children in Zambia after MDA

**DOI:** 10.1371/journal.pone.0189400

**Published:** 2017-12-11

**Authors:** Megan J. Hessler, Austin Cyrs, Steven C. Krenzke, El Shaimaa Mahmoud, Chummy Sikasunge, James Mwansa, Nilanjan Lodh

**Affiliations:** 1 Department of Clinical Laboratory Science, Marquette University, Milwaukee, Wisconsin, United States of America; 2 Department of Biology, Marquette University, Milwaukee, Wisconsin, United States of America; 3 Department of Para-clinical Studies, The University of Zambia, Lusaka, Zambia; 4 University Teaching Hospital, The University of Zambia, Lusaka, Zambia; George Washington University School of Medicine and Health Sciences, UNITED STATES

## Abstract

Schistosomiasis is one of the major Neglected Tropical Diseases (NTDs) in sub-Saharan Africa. In sub-Saharan Africa, two major human schistosome species namely *Schistosoma mansoni* and *S*. *haematobium* often occur sympatrically largely affecting children. Recognizing the public health impact of Schistosomiasis, the World Health Organization (WHO) is urging member states to regularly treat at least 75% and up to 100%, of all school-aged children at risk of morbidity. For control strategies based on targeted mass drug administration (MDA) to succeed it is essential to have a simple and sensitive test for monitoring the success of these interventions. Current available diagnostic tests, such as egg detection in stool by Kato-Katz (KK) for *S*. *mansoni* and detection of eggs or blood (hematuria) in urine for *S*. *haematobium* have reduced sensitivity in low intensity settings. The objective of the study was to evaluate active single or duo schistosome infections in school children following MDA using molecular diagnostics (PCR) on filtered urine samples and comparing that against traditional diagnostic tests. This cross-sectional study was conducted among 111 school children aged 7–15 years in Chongwe and Siavonga Districts in Zambia. Species-specific cell-free repeat DNA fragment were amplified from 111 filtered urine samples. Our approach detected eight times more positive cases (total 77) than by KK (9) for *S*. *mansoni* and six times more (total 72) than by hematuria (11) for *S*. *haematobium* and even more against urine filtration (77 compared to only 6). The same pattern was observed when stratified for age group and sex specific analysis with 100% sensitivity and specificity devoid of any cross amplification. In addition, 69 individuals (62%) were co-infected by both parasites. We have demonstrated a significantly higher prevalence of both species than indicated by the traditional tests and the persistent maintenance of reservoir of infection after MDA. Our approach is an effective means of detecting low intensity infection, which will enhance the effectiveness of surveillance and assess the impact of MDA control programs against schistosomiasis.

## Introduction

Schistosomiasis in Africa is an ongoing public health problem, which in recent times has attracted a major campaign to control the disease. In 2014, approximately 91% of people estimated to require treatment for schistosomiasis lived in the African region [1]. Children are particularly vulnerable to schistosome infections and are likely to be the main carriers of infection due to their large egg output and increased water contact [2]. Recognizing the public health impact of schistosomiasis on school-age children, the World Health Organization (WHO) is urging member states to regularly treat at least 75% and up to 100%, of all school-aged children at risk of morbidity [3]. Forty three million school-age children received treatment in 2014 comprising >83% of the total number of people treated [1]. As the control programs become more and more effective in reducing the parasite burden in children, the issue of diagnostic sensitivity will become more critical in the assessment of program effectiveness. It is important to determine if these children can still infect snails (vector) and keep the transmission going. For mass drug administration (MDA) to succeed, these reservoirs of infection must be diagnosed and the diagnostic method to do so should be one that is easy to operate, sensitive and accurate.

The diagnostic problem for schistosomiasis is exacerbated by the fact that in Africa, the two-parasite species, *Schistosoma mansoni* and *S*. *haematobium* are often sympatric and concurrent infection is debilitating [4]. Because different organ systems are involved, the pathological implications of single or mixed infections are different, it is therefore important to differentiate accurately between them. Present clinical studies are often unable to detect light infections as well as sympatric, multiple parasite infection due to lack of sensitivity and specificity of current parasitological and proposed point of care (POC) techniques [5]. Searching for parasite eggs in light intensity infections is difficult and more often than not, such infections are misdiagnosed [6].

Current available diagnostic tools, such as egg detection in stool by Kato-Katz (KK) for *S*. *mansoni* and detection of eggs or blood (hematuria) in urine for *S*. *haematobium* lack sensitivity in low intensity settings [5, 7, 8]. Our previous work successfully detected schistosome specific DNA in *S*. *mansoni* [8] and *S*. *haematobium* [7] from filtered urine using polymerase chain reaction (PCR) with high sensitivity (true positive) and specificity (true negative) with no cross-reactivity with other related parasites. We also demonstrated that it is possible to detect DNA specific to both of these two schistosome parasites from a single source of urine, thus simplifying the collection and performance of tests that are more sensitive and more specific than the standard diagnostic tests [9]. It is important to note that the presence of DNA in urine indicates a viable infection. The test is superior in sensitivity and specificity to KK and hematuria [7–9].

Our recently developed diagnostic test targets mass treatment program and thus addresses the issues of sensitivity and specificity. It also addresses logistical problems associated with sample collection and handling in situations where stool samples are needed. To evaluate the control or intervention programs, more sensitive and specific tests need to be introduced. We reiterate here as campaigns progress, infections become less severe and the tests will become less effective, missing detection of asymptomatic carriers who are the source of continued transmission. To address this, we tested our diagnostic technique in Zambia in an on-going schistosomiasis control intervention (mass and targeted chemotherapy distributed through schools). Therefore, a method was devised that avoids working with fecal samples in the detection of *S*. *mansoni* infections in school going children in two of the Schistosomiasis endemic districts (Chongwe and Siavonga) in Zambia. Hence the purpose of this study was to evaluate active single or duo schistosome infection in school children after MDA with highly sensitive and specific species-specific repeat DNA detection from filtered urine samples and comparing that against traditional diagnostic tests.

## Materials and methods

### Study design and sample population

A cross-sectional study was conducted in two districts, namely, Chongwe district in Lusaka Province and Siavonga district in Southern Province. Chongwe district is located near the highly meandering Chongwe River while Siavonga district is along the shores of Lake Kariba. These study districts were selected based on previous mapping results, which indicated endemicity for both schistosome species. The selected districts thus were and are part of the on-going annual MDA and general infection on intensity was therefore expected to be low. The study population was comprised of 111 school going children aged 7–15 years old (60 female and 50 male students; Table 1). For one children, the sex was not recorded, but was included in the analysis (S1 Data). Participants were recruited in July 2016, one (1) month after MDA with praziquantel given at 40mg/kg. Samples were collected from participants after obtaining written informed consent from parents or guardians and a verbal assent from minors. As this was a cross sectional study, there were no drop outs.

**Table 1:** Demographic characteristics of the study population.

Age group			
Sex	7–12 years	13–15 years	Total
Male	31	19	50
Female	44	16	60
**Total**	75	35	**110**^¶^

### Data (sample) collection

All participants provided both urine and stool samples after the purpose of the study was explained to them and consent obtained. Urine specimens were collected in plastic cups, and then numbered and referenced across all filtered urine samples. Each student volunteer was then provided with a second cup for stool. The same reference number was used for all specimens from a single volunteer. The number assigned to the volunteer was transferred on the filter paper prior to urine filtration. The information that was made available to the researcher in the USA was only age, sex and locality of each sample. All personal reference was secured and retained in Zambia. The study was approved by institutional review board (IRB) of Marquette University, USA (IRB # HR-3116) and by ERES Converge, Zambia (IRB # 2016-Apr-002).

### Parasitological tests

Stool samples were processed and examined for presence of *S*. *mansoni* egg by using WHO standard KK kit (WHO, Geneva, Switzerland). Two stool smears per sample were examined by KK [10]. All urine samples were tested for hematuria for *S*. *haematobium* by Multistix (Siemens Healthcare Diagnostics Inc., Tarrytown, NY, USA) and urine filtration (Sterlitech, Washington, USA) for presence of *S*. *haematobium* eggs. In addition, 40 ml of urine were filtered through Whatman # 3 filter paper, air dried and then packed individually in Ziploc bags shipped to Milwaukee, Wisconsin USA. This was a research project and the technique is intended to be introduced in country with subsequent proposals and recommended to the National control program.

### DNA extraction from filtered urine

DNA from filtered urine samples were extracted by QIAmp DNeasy^®^ Blood and Tissue Kit (Qiagen, Hilden, Germany). The filter paper quadrant used for draining the urine was used to punch 15 holes (~1mm diameter) by regular paper punch. The paper punch and scissor was sterilized by washing with 10% bleach and nuclease-free-water after every use for minimizing any chance of contamination [9]. The paper discs were added to nuclease-free-water in 2 ml Eppendorf tubes. These were then heat shocked at 95°C for 10 minutes. After heat shock, the tubes were left on a shaker at 150 rpm for overnight.

The next morning, tubes were removed from the shaker and centrifuged at 8,000 x g for 1 minute. The liquid in tube was transferred into a QIAmp 2 ml column tube. Then DNA was extracted according to the manufacturer’s protocol. All extracted DNA was quantified by using Nanodrop (Thermo Scientific, Wilmington, DE, USA) to determine the concentration of the extracted DNA after which all the extracts were stored at -20°C until use.

### PCR amplification of schistosome species

Species-specific primers [9] were used (Table 2) to amplify cell-free repeat DNA fragments from filtered urine samples to confirm the presence of either *S*. *mansoni* or *S*. *haematobium* or both. For every reaction, three different controls were used: 1) *S*. *mansoni* and *S*. *haematobium* genomic DNA (BEI Resources, Manassas, VA, USA) as positive control; 2) extracted DNA from urine collected in-house as negative control; and 3) nuclease-free-water as water control (Sigma-Aldrich, St. Louis, MO, USA). A commercially purchased Mastermix (New England Biolabs, Ipswich, MA, USA), 10μM forward and reverse primers, magnesium, and nuclease-free-water (Sigma-Aldrich, St. Louis, MO, USA) was used for a 20μl PCR reaction. PCR amplification took place in an automated thermocycler with the following settings. For amplification of *S*. *mansoni*, the denaturation at 95°C for 10 minutes was followed by annealing process of 57°C for 90 seconds, which was repeated 35 times and ended with an extension at 72°C for 10 minutes. For the amplification of *S*. *haematobium*, the steps were same for denaturation expect that the annealing process was set at 56°C for 90 seconds and final extension of 72°C for 1 minute.

**Table 2:** Primer sets used for species-specific cell-free repeat DNA amplification of *Schistosoma mansoni* and *S*. *haematobium* by PCR method.

Schistosome parasite	Oligonucleotide name	Oligonucleotide sequence	Reference
*Schistosoma mansoni*	SmPF	5' GAT CTG AAT CCG ACC AAC CG 3'	Lodh et al., 2014
	SmPR	5' ATA TTA ACG CCC ACG CTC TC 3'	
*Schistosoma haematobium*	Sh*Dra1*F	5′ TCA CAA CGA TAC GAC CAA C 3′	Lodh et al., 2014
	Sh*Dra1*R	5′ GAT CTC ACC TAT CAG ACG AAA C 3′	

After amplification, gel electrophoresis was run to visualize the amplified species-specific DNA fragments. A 2% agarose gel stained with SYBR Green (Thermo Scientific, Waltham, MA, USA) was used. A 50bp reference ladder (New England Biolabs, Ipswich, MA, USA) was used for size comparison of amplified fragment. All agarose gels were visualized in Azure C200 gel documentation system (Azure Biosystems, Dublin, CA, USA).

### Statistical analyses

The sensitivity and specificity of PCR amplification for both species were compared against KK, urine filtration and hematuria using MedCalc 12.4.0 (MedCalc Software, Ostend, Belgium). The data was also stratified for two different age groups (Group A: 7–12 years and Group B: 13-15yeras) and sex (female and male) for determining the efficacy of different diagnostic approaches. Results were converted to numerical values (1 = positive and 0 = negative) for performing the statistical analysis. The positive and negative assessment of each sample was based on following predictions.

- If KK +, then sure presence of *S*. *mansoni* infection
- If *S*. *mansoni* PCR +, then assume presence of *S*. *mansoni* infection
- *S*. *mansoni* True Positive (TP): If KK + and *S*. *mansoni* PCR +
- *S*. *mansoni* True Negative (TN): If KK − and *S*. *mansoni* PCR −
- If urine filtration +, then sure presence of *S*. *haematobium* infection
- If *S*. *haematobium* PCR +, then assume presence of *S*. *haematobium* infection
- *S*. *haematobium* True Positive (TP): If urine filtration + and *S*. *haematobium* PCR +
- *S*. *haematobium* True Negative (TN): If urine filtration − and *S*. *haematobium* PCR −

Disease prevalence was calculated based on number of positive cases by each diagnostic test against the total number of samples were evaluated. Agreement statistics were calculated by measuring Kappa value and Bowker’s Symmetry by JMP 12 (JMP^®^ v12, SAS Institute Inc., Cary, North Carolina, USA) to establish the agreement between two diagnostic tests. Kappa coefficient range (-1–0 –+1) determined the agreement between two tests, where +1 = complete agreement, 0 = marginal agreement and -1 = no agreement. Bowker’s symmetry determined the lack of agreement (JMP^®^ v9, SAS Institute Inc., Cary, North Carolina) between two tests at a time. This test checked for symmetry in 2-way tables and the test decision was based on *X*^*2*^ approximation of the distribution of the test statistic [11].

## Results

### Detection of *Schistosoma mansoni* by KK and PCR

**Fig 1:**
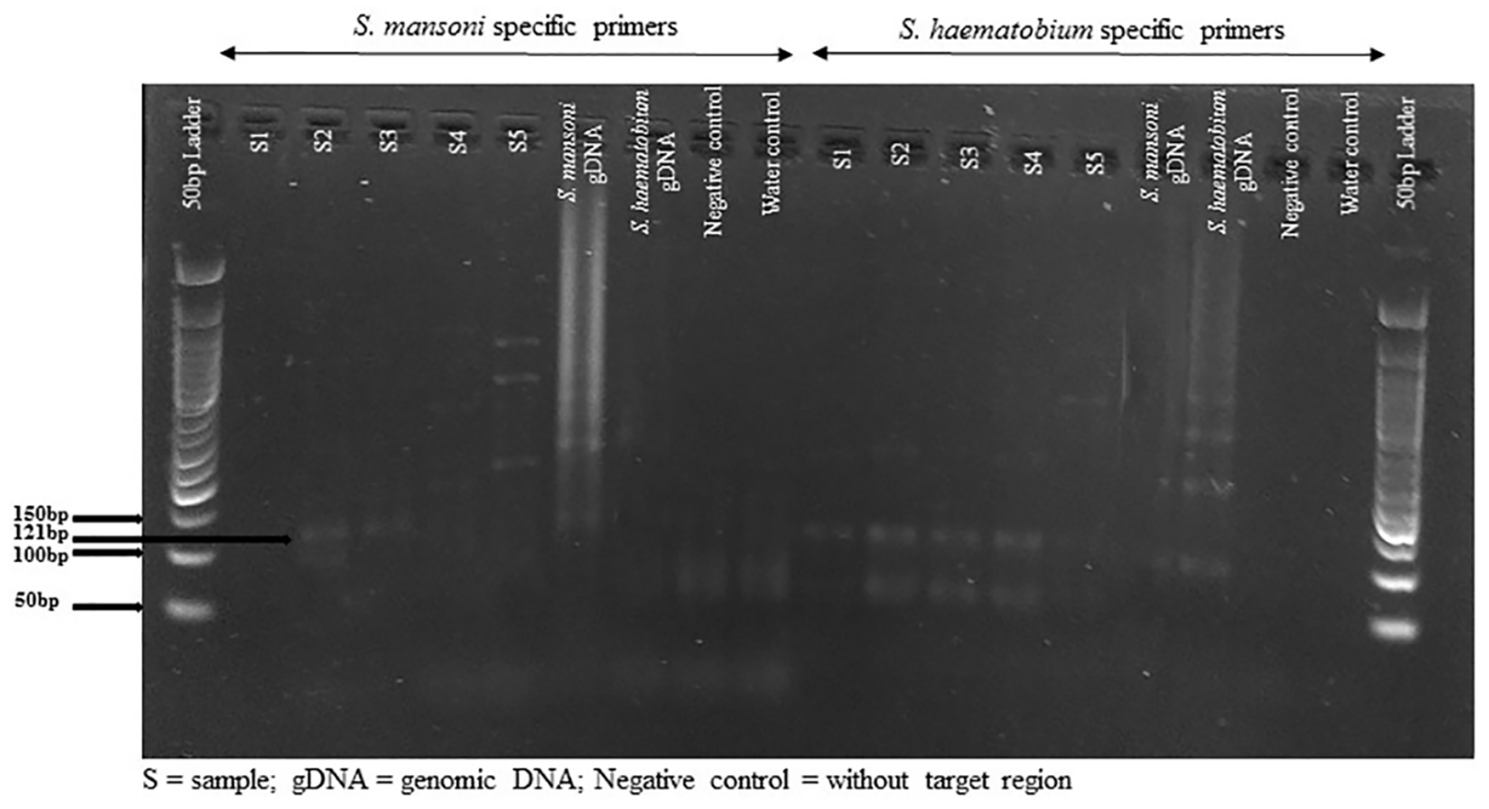
Agarose gel image of the repeat fragment amplicons for both *S*. *mansoni* and *S*. *haematobium* with species-specific primers.

The purpose of this research was to determine the effectiveness of detecting small amount of parasite repeat DNA fragments in urine samples from school children infected with *S*. *mansoni* or *S*. *haematobium*, or both after MDA (Fig 1). Conventional PCR performed in the laboratory was compared to detection methods used in the field, such as KK for stool samples and hematuria and urine filtration for urine examination.

The comparison of KK and PCR in the detection of *S*. *mansoni* infection was most notably distinguished when dealing with low-level infections (no egg is found in the stool sample) and asymptomatic infections. Of the 111 samples that were tested, only 9 were positive by both KK and PCR, whereas 77 were positive by PCR (Table 3). Overall 25 were negative by both KK and PCR (Table 3). There were no false positives produced by either method. PCR was the better indicator of disease prevalence (78% vs. 8%; Table 4). With a sensitivity of 100% compared to only 11% for KK (Table 4). The implication of negative infection due to KK test [Negative Predictive Value (NPV): 25%, Table 4] was a great underestimate of actual number of school children that were infected or had retained infection (NPV: 100%; Table 4). A significantly low level of agreement was detected between KK and PCR (Kappa = 0.05; 95% CI: 0.01–0.09; Table 5). The Bowker Symmetry was significantly different from random for the comparison, indicating that PCR will detect more positive infection than the ones positive by KK due to its higher sensitivity (Table 5).

**Table 3:** The comparison of diagnostic evaluation of 111 field collected stool and urine samples for both *Schistosoma mansoni* and *S*. *haematobium*. *S*. *mansoni* egg presence in stool was evaluated by Kato-Katz (KK) and *S*. *haematobium* was evaluated by measuring hematuria (sign of blood) and by urine filtration for egg presence in urine. The filtered urine samples were evaluated for presence of both species by amplifying species-specific repeat DNA fragment.

***Schistosoma mansoni***		
N = 111 samples evaluated	KK negative	KK positive
Urine PCR negative	25 (23%)	0
Urine PCR positive	**77 (69%)**	9 (8%)
***Schistosoma haematobium***		
N = 111 samples evaluated	Hematuria negative	Hematuria positive
Urine PCR negative	28 (25%)	0
Urine PCR positive	**72 (65%)**	11 (10%)
N = 111 samples evaluated	Urine filtration negative	Urine filtration positive
Urine PCR negative	28 (25%)	0
Urine PCR positive	**77 (69.5%)**	6 (5.5%)

**Table 4:** Qualitative analysis to determine disease prevalence, sensitivity, specificity, and predictive values of Kato-Katz (KK), hematuria, urine filtration and species-specific DNA amplification via PCR for identifying single or mixed schistosome infection from school children after MDA from Zambia.

Diagnostic Test	Disease Prevalence*	Sensitivity (95% CI)	Specificity (95% CI)	Positive Predictive Value (PPV)	Negative Predictive Value (NPV)
KK for *S*. *mansoni*	8%	11% (5%–19%)	100% (86%–100%)	100%	25%
PCR for *S*. *mansoni*	78%	100% (96%–100%)	100% (86%–100%)	100%	100%
Hematuria for *S*. *haematobium*	10%	13% (7%–23%)	100% (88%–100%)	100%	28%
Urine filtration for *S*. *haematobium*	5%	7% (3%–15%)	100% (88%–100%)	100%	27%
PCR for *S*. *haematobium*	75%	100% (96%–100%)	100% (88%–100%)	100%	100%

*Disease prevalence = proportion of positive infection by each test out of total number of samples were evaluated

**Table 5:** Agreement statistics estimation (Kappa coefficient and Bowker symmetry test) comparing species-specific DNA amplification method for both Schistosome species against Kato-Katz (KK), hematuria and urine filtration.

Comparison of diagnostic tests	Kappa coefficient		Bowker’s symmetry test^$^	
	Degree of agreement	95% CI	Symmetry of disagreement	P value^ψ^
*Schistosoma mansoni*	0.05	0.01–0.09	77	0.0001*
KK vs. urine PCR				
*Schistosoma haematobium* Hematuria vs. urine PCR	0.07	0.02–0.19	72	0.0001*
Urine filtration vs. urine PCR	0.04	0.01–0.07	77	0.0001*

^$^Bowker’s Symmetry test = this test checks for symmetry in 2-way tables and the test decision is based on a *X*^2^ approximation of the distribution of the test statistic

^ψ^ = α level was set at 0.05

* = Significant

### Detection of *Schistosoma haematobium* by hematuria, urine filtration and PCR

In the field, dipstick (to detect hematuria) and urine filtration (to detect *S*. *haematobium* egg) were used to determine the presence of *S*. *haematobium* infection. A PCR was performed to amplify the *S*. *haematobium* specific DNA from filtered urine sample. Again, PCR was the best method for detecting *S*. *haematobium* infection. Hematuria exhibited far more false negative (FN) cases (72) than PCR (none: Table 3). There were 28 samples negative by both hematuria and PCR, and 11 samples were positive by both methods (Table 3). This was expected as hematuria is a non-specific indicator of *S*. *haematobium* infection. Urine filtration yielded even more FN (77) than hematuria did (Table 3) compared to PCR (none). Also, PCR was the best method 100% sensitive compared to hematuria (13%) and urine filtration (7%; Table 4). The Kappa agreement statistics indicated that PCR performed significantly better in detecting *S*. *haematobium* infections than hematuria (Kappa = 0.07; 95% CI: 0.02–0.19; Table 5). The symmetry of disagreement was also significantly higher (72; P = 0.0001), which indicated that PCR scored more positives, especially when infection was asymptomatic and cannot be determined by hematuria (Table 4). Similarly, there was a significant low degree of agreement between urine filtration and PCR (Kappa = 0.04; 95% CI: 0.01–0.07; Table 5). The Bowker’s symmetry was higher when comparing these two methods (77; P = 0.0001; Table 5).

### Duo species detection by parasitological and molecular diagnostic assay

This study found school children infected with both schistosome species detected from extracted DNA from a single urine specimen. A total of 69 children out of 111 children evaluated were found to have mixed infection. The mixed infection was comparatively much higher for PCR detection of both *S*. *mansoni* and *S*. *haematobium* (62%) than KK, hematuria and urine filtration combined (Table 6). Single infection for *S*. *mansoni* was at 17 by PCR whereas KK detected only 7. For *S*. *haematobium* it was 14 by PCR and only 9 and 4 by hematuria and urine filtration, respectively (Table 6). An unexpectedly four times more children who did not show any signs of being infected using parasitological methods came out positive for both species by species-specific repeat DNA detection (Table 6).

**Table 6:** Detection of single or mixed *S*. *mansoni* and *S*. *haematobium* infection by KK, hematuria, urine filtration and PCR. Test specific for each species has been mentioned.

Diagnostic tests	Single infection (by only one test)	Mixed infection (by both species^¶^)	Total # of infection
KK (*S*. *mansoni*)	7	2 (2%)	102
Hematuria (*S*. *haematobium*)	9	2 (2%)	100
Urine filtration (*S*. *haematobium*)	4	2 (2%)	105
*S*. *mansoni* PCR	17	**69 (62%)**	25
*S*. *haematobium* PCR	14	**69 (62%)**	28

^¶^ = by KK and hematuria or by KK and urine filtration or by all three tests.

### Age and gender-specific detection

**Fig 2:**
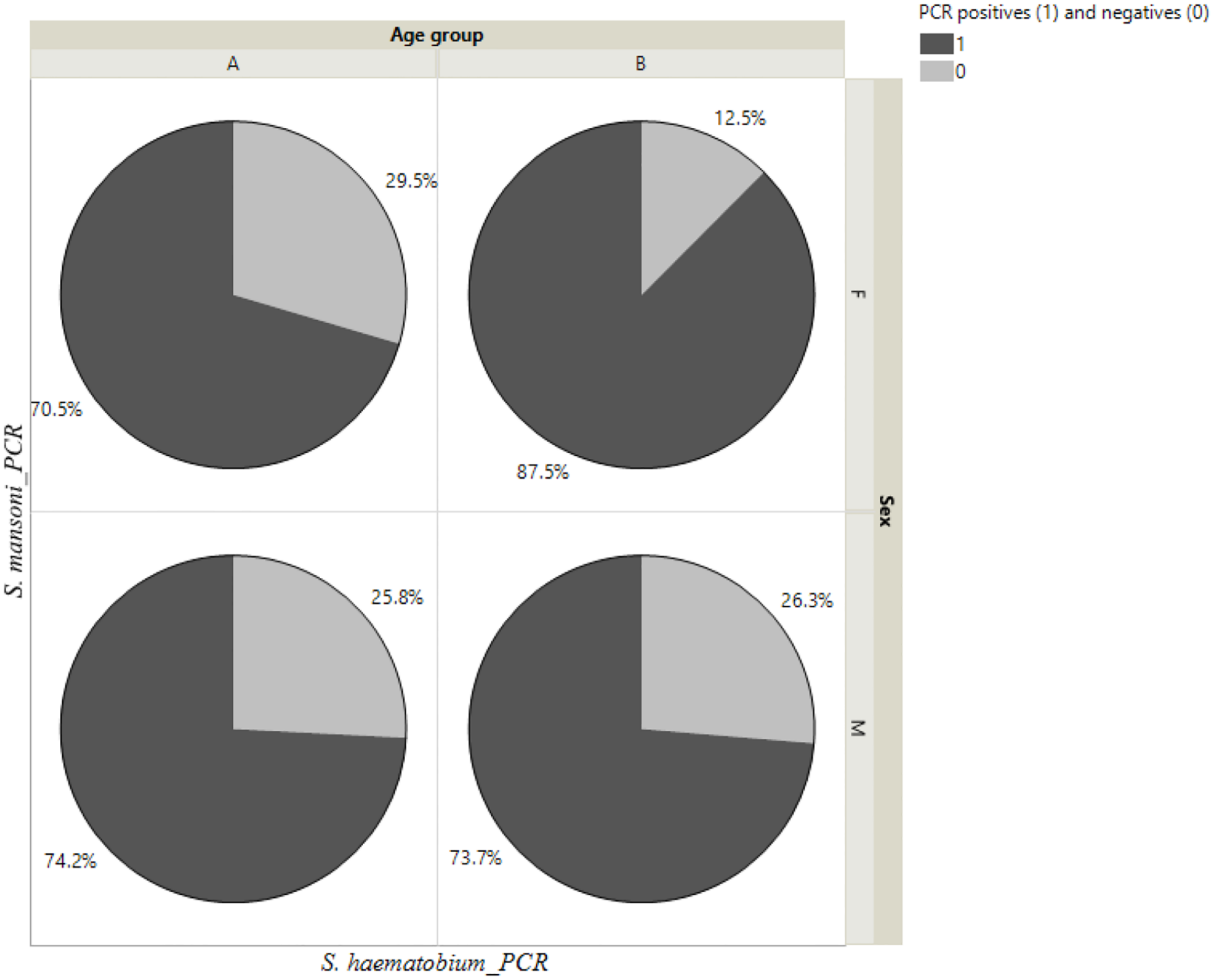
Detection of dual infection (both *S*. *mansoni* and *S*. *haematobium*) by species-specific repeat DNA amplification from single urine sample. The dual infection for both species is categorized into two different age groups (Group A: 7–12 years and Group B: 13–15 years) and for both female and male participants.

All the above-mentioned tests were evaluated for both female and male participants and for two age groups (Group A: 7–12 years and Group B: 13–15 years). PCR proved to be the best indicator of positive infection in female participants for both pre-teen and teen-age groups at 70% and 87%, respectively when compared for mixed infection (Fig 2). The same outcome was also evident for male school children with both age groups 74% and 74% (Fig 2). Parasite DNA detection effectively identified positive infection irrespective of sex and age.

## Discussion

We have demonstrated a significantly higher prevalence of *S*. *mansoni* or *S*. *haematobium* and/or both in school children than indicated by the classical examination of urine or stool after MDA. Our findings have shown that detection of cell-free repeat DNA via PCR is at least six to eight times more sensitive than any of the parasitological methods, KK, hematuria and urine filtration. It can be seen clearly from our results that after MDA, use of hematuria and egg detection missed the detection of the actual *S*. *haematobium* infections. Definitive tests have depended on detecting parasite eggs in the urine and stool, but with low intensity infections especially after MDA these tests have been shown to have low sensitivity. This is prominently evident by our current findings (Table 4). More interestingly, the revelation of 62% of school children with mixed infection (Table 6) after treatment indicates the distribution of both species and their snail host, as the pathogenicity and disease outcome is significantly different for *S*. *mansoni* and *S*. *haematobium* [12]. Our findings have shown that PCR can detect a true positive or a true negative even when the infection load is lower as may be the case after MDA. With drug treatment, infections become less severe and the traditional tests will become less effective, missing detection of asymptomatic carriers who might be the source of continued transmission [13]. Highly sensitive species-specific DNA detection via PCR is beneficial, because if the schistosomes can be more accurately diagnosed, then the drug that treats schistosomiasis, Praziquantel (PZQ), can be used more efficiently in order to prevent any future drug resistance.

The drug PZQ is unique. It is the only drug that effectively treats the three-major human schistosomes including *S*. *japonicum* [14–16]. PZQ is a widely used drug against schistosome species and its usage is likely to grow in the near future [17]. Although there is no clinically relevant evidence of PZQ resistance [17], but it still remains a threat. So, it would be wise to consider adequate monitoring of current MDA programs. It is important to decipher the frequency of re-infection and remaining infection after treatment [18]. The long-term success of MDA is dependent on the ability of health authorities to diagnose persistent low intensity infections that persist even after treatment has occurred [19]. People with low parasitaemia still pass viable schistosome eggs out in the urine and stool and the hatching miracidia are efficient in locating the intermediate host snails [20] and maintaining the cycle. International agencies are now attempting to introduce targeted therapy in many, resource poor areas in Africa where schistosomiasis is prevalent [21]. The main problem in sustaining these efforts is with the diagnostic tests available to detect infection. Thus, with re-infection and persistent infection in children when treatment is only partially effective, many infected individuals usually remain with undiagnosed infection. Therefore, sensitive and accurate tests such as the one used in this study that can be used to detect for both sympatric species from one sample across different age group and irrespective of gender are urgently needed (Fig 1).

Detection of parasite specific DNA in urine or bodily fluids indicates the presence of the actual parasite even when eggs are not always detectable [22]. We have successfully detected schistosome specific DNA in *S*. *haematobium* [7] and *S*. *mansoni* [8] from filtered urine and always showed high sensitivity and specificity with no cross-reactivity with other related helminths. We have also demonstrated that it is possible to detect DNA specific to both schistosome parasites from a single source of urine, thus simplifying the collection and performance of tests that are more sensitive and more specific than the standard diagnostic tests [9]. Our current findings were consistent with our previous findings and would be more useful for targeted therapy and a successful control intervention.

We acknowledge however, the limitations of the small sample size used in our study to demonstrate the efficacy of different diagnostic tests after drug treatment. A large sample size coupled with the use of other tests, such as the Circulating Cathodic Antigen test (CCA) for *S*. *mansoni* and Circulating Anodic Antigen test (CAA) for *S*. *haematobium* would probably have given a more informative or explanatory result. The usage of PCR as a diagnostic test also has technical limitation. PCR amplification requires the use of a thermocycler for amplification and electrophoresis equipment for visualization. These are difficult to implement in resource constraint areas and under field conditions. The need for the equipment can be replaced in the field using loop-mediated isothermal amplification (LAMP) [23] or recombinase polymerase amplification (RPA) [24]. LAMP procedure uses three sets of primers to amplify six regions of the target DNA. Amplification of the DNA occurs independently of a thermocycler, and will change turbidity if the reaction is positive [25]. While the concept of adapting DNA amplification-based diagnosis/monitoring to field laboratories in Africa will likely require a quantum leap in diagnostic facilities, its initial establishment may benefit from sophistication.

Therefore, if this species-specific PCR was to be developed in a LAMP form, it would make this diagnostic technique more affordable, quick, specific and simple to use even in the field. This can be used as an integrated part of surveillance program to measure the effectiveness of control intervention. Innovative diagnostic tools for the “end-game” of control interventions are warranted in the control of schistosomiasis and PCR and LAMP based methods cannot be ruled out. Our study has demonstrated that without stool examination, urine filtered through filter papers could be used to concurrently diagnose both *S*. *mansoni* and *S*. *haematobium* species.

## Supporting information

S1 Data Data for all 111 samples that has been used in the study. (XLSX)
